# Computer-Assisted Photo Identification Outperforms Visible Implant Elastomers in an Endangered Salamander, *Eurycea tonkawae*

**DOI:** 10.1371/journal.pone.0059424

**Published:** 2013-03-21

**Authors:** Nathan F. Bendik, Thomas A. Morrison, Andrew G. Gluesenkamp, Mark S. Sanders, Lisa J. O’Donnell

**Affiliations:** 1 Watershed Protection Department, City of Austin, Austin, Texas, United States of America; 2 Wyoming Cooperative Fish and Wildlife Research Unit, Department of Zoology and Physiology, University of Wyoming, Laramie, Wyoming, United States of America; 3 Texas Parks and Wildlife Department, Austin, Texas, United States of America; 4 Balcones Canyonlands Preserve, City of Austin, Austin, Texas, United States of America; University of Western Australia, Australia

## Abstract

Despite recognition that nearly one-third of the 6300 amphibian species are threatened with extinction, our understanding of the general ecology and population status of many amphibians is relatively poor. A widely-used method for monitoring amphibians involves injecting captured individuals with unique combinations of colored visible implant elastomer (VIE). We compared VIE identification to a less-invasive method – computer-assisted photographic identification (photoID) – in endangered Jollyville Plateau salamanders (*Eurycea tonkawae*), a species with a known range limited to eight stream drainages in central Texas. We based photoID on the unique pigmentation patterns on the dorsal head region of 1215 individual salamanders using identification software Wild-ID. We compared the performance of photoID methods to VIEs using both ‘high-quality’ and ‘low-quality’ images, which were taken using two different camera types and technologies. For high-quality images, the photoID method had a false rejection rate of 0.76% compared to 1.90% for VIEs. Using a comparable dataset of lower-quality images, the false rejection rate was much higher (15.9%). Photo matching scores were negatively correlated with time between captures, suggesting that evolving natural marks could increase misidentification rates in longer term capture-recapture studies. Our study demonstrates the utility of large-scale capture-recapture using photo identification methods for *Eurycea* and other species with stable natural marks that can be reliably photographed.

## Introduction

Global declines in amphibian abundance and diversity have received considerable attention in recent years because of the alarming rates of loss, particularly relative to other major taxonomic groups [1]. In some cases, entire populations or even species have disappeared in the course of a few years [2]. The swiftness of these declines, and our limited understanding of the general ecology and population status of many species (22.5% of amphibians are considered ‘data deficient’ in the IUCN species status classification [1]) provide strong motivation to develop more rapid, reliable and non-invasive methods for monitoring populations in the hopes of identifying major threats and developing conservation remedies [3].

Many of the most common and powerful methods for collecting demographic and movement data (e.g. capture-recapture) involve physically capturing, handling and marking study organisms and re-identifying them during subsequent surveys [4]. However, amphibians are often challenging to mark due to their darkly pigmented, sensitive skin and their small size. Common methods for tagging amphibians include tattooing, branding, toe-clipping, passive integrated transponder tagging and implanting colored elastomers under the skin of captured animals [5]. This latter method – visual implant elastomers (VIE) – has become popular because it can be used to identify both larval and adult stages (and metamorphosis between the two), though observer identification ability and mark retention are significant issues [6].

Photographic identification (photoID) is an increasingly popular technique used in capture-recapture studies. Advances in digital image analysis tools and pattern recognition algorithms have accelerated the application of photoID to a wide range of species with natural marking patterns, including amphibians [7], [8], [9], reptiles [10], terrestrial mammals [11] and fishes [12], among others (for review, see Table 1 in [13]). While computer-assisted photoID is only useful in species with variable natural markings, it has the advantage of being relatively inexpensive (entailing only a digital camera and computer), requiring only basic technical expertise beyond the development of image analysis tools, and allowing large numbers of individuals to be re-identified. In the context of monitoring populations of endangered or threatened species, developing survey methods that minimize the potential handling effects and welfare concerns related to animal capture is paramount [14].

**Table 1:** Summary of datasets used in false rejection rate (FRR) and false acceptance rate (FAR) error calculations.

Photo dataset	Years	#Indiv.	#Captures	#Matching photo pairs (recaptures)	Error Rates					
					VIE FRR	VIE FAR	VIE error sample size^a^	Photo FRR	Photo FAR	PhotoID error sample size^a^
Low-quality	2007	473	965	896	0.0190 (0.0003)	0.0178 (0.0002)	554	0.1591 (0.0001)	0.0132 (0.0004)	264
High-quality	2008–2010	742	1367	1090				0.0076 (0.0002)	0.0000 (0.0000)	356

Standard errors are included in parentheses.

a per iteration.

Regardless of identification technique, if markings change over time or are relatively invariable across individuals, misidentification errors are likely. These errors are problematic in capture-recapture studies because they violate the assumption that individuals are correctly identified; when ignored, errors can bias demographic parameter estimates [15], [16]. Often, studies do not assess or report probability of making misidentification errors [13], which makes it difficult to contrast the relative performance of one method against another (though see [5], [8]).

Jollyville Plateau salamanders (*Eurycea tonkawae*) are endemic to central Texas and one of four closely-related species that are currently proposed for protection under the US Endangered Species Act [17]. Here, we test the performance of a computer-assisted photoID system and a VIE system in a wild population of *E. tonkawae* using available data on individuals captured over a four-year period. Our goals are to (1) illustrate the advantages and limitations of using either of these methods, with the bottom line being the ability to accurately identify individual salamanders; (2) determine the accuracy of automated photo matching using both ‘high’ and ‘low-quality’ photographs; and (3) provide tips and example code for determining whether photoID can be applied to a novel organism and/or research setting.

## Materials and Methods

### Study Organism and Study Site

*Eurycea tonkawae* is a small (total length typically <75 mm) neotenic salamander endemic to springs, spring-fed headwater streams, and wet caves in northwest Austin, Texas and surrounding areas, with a total range of <110 km^2^ [18], [19]. Our study site includes a small, occasionally intermittent spring (Lanier Spring) and adjacent stream bed (Bull Creek) within the City of Austin’s Balcones Canyonland Preserve, Travis County, Texas. Salamanders were captured with aquarium nets using a drive survey technique that exposed all available surface cover (rocks, leaves, algae) within the study area (64 m^2^). Sampling consisted of 42 total capture-recapture surveys consisting of 14 primary periods with consecutive 3-day secondary periods between 2007 and 2010.

### Visible Implant Elastomers

Salamanders >16 mm snout-vent length were anaesthetized in a solution of 0.25 g Tricaine S (MS-222)/L of naturally-buffered spring water and then marked using VIE tags (Northwest Marine Technology Inc., Shaw Island, Washington). Sterile 28-gauge syringes were used to inject small amounts (2–20 µL) of elastomer just underneath the skin to form a bead. Each salamander was given three to four unique VIE tags using a combination of seven different colors in five locations on the body. Captured and recaptured salamanders were photographed (details below) initially so that their natural marks could serve as a secondary mark to check our VIE identifications. Using this photographic database, each recaptured salamander was compared to a previous capture of the same individual (based on VIE identification) by manually (i.e., ‘by-eye’) comparing their natural marks (melanophore and iridophore pigmentation patterns). This process allowed us to confirm or correct most VIE identifications, although a small number (<2%) of salamanders with missing or unidentifiable VIE tags were impossible to match manually. When referring to the identification techniques used throughout this paper, we use the phrase VIE as shorthand to refer to the entire VIE-based identification system, which includes this manual photo-matching validation step. Thus, any errors reported as ‘VIE’ errors occurred after both visually checking VIE tags and manually checking photos for confirmation of a match.

### Animal Welfare

The anaesthetization procedure was reviewed and approved by a veterinary professional (Chris Sanders, DVM). The VIE tagging procedures we used were approved by the IACUC of the University of Texas at Austin for a separate study designed to evaluate the effects of marking on individual growth and performance of two closely related species, *Eurycea sosorum* and *E. nana*. (IACUC protocol #07072701). The co-author (AGG) of that study also conducted and oversaw all VIE tagging for the present study. Additionally, care was taken to ensure animals being held were maintained at the ambient water temperature by keeping them in flow-through mesh boxes within their habitat during processing or in containers with frequent water changes. The data we present here were not collected primarily for this study, but capture-recapture (and associated VIE tagging) was carried out by the City of Austin Watershed Protection Department for the purpose of understanding the population dynamics of *E. tonkawae.* Field collections were conducted under Texas Parks and Wildlife Scientific Permit SPR-1005-1515.

### Computer-assisted PhotoID

The second identification technique involved computer-assisted salamander identification using digital photographs of pigmentation patterns in the dorsal head regions of individuals. Salamanders were photographed in a shallow water-filled tray with a white background initially using a Nikon Coolpix E995 ‘point-and-shoot’ camera (for 24 surveys in 2007) but then were later photographed using an advanced DSLR system: Nikon D80, 90 mm macro lens and two close-up flashes (for 18 surveys from 2008–2010). Photos were taken handheld, with an effort to ensure that the focal plane and salamander dorsum were parallel. DSLR camera settings included an aperture of f/16 or smaller, a shutter speed of 1/160 s or faster, and high-quality JPEG settings.

**Figure 1:**
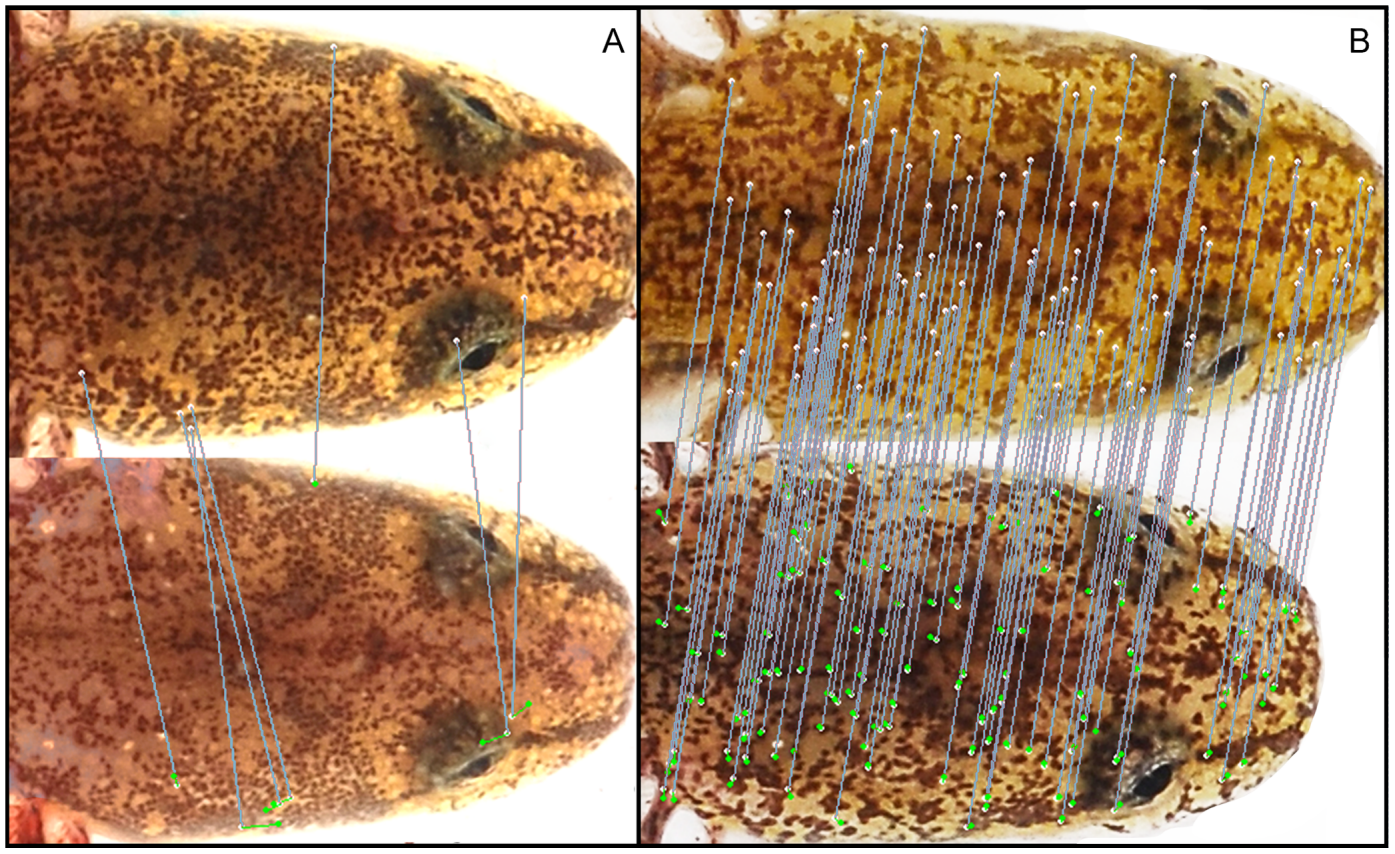
Head pattern recognition in *Eurycea tonkawae*. Pair of images from (A) two different individuals and (B) the same individual one year apart. Lines connect matching SIFT features.

After cropping each photograph to include only the head, all photographs in our database were compared and matched using open-source pattern identification software called ‘Wild-ID’ (ver. 1.0.1 [13]; http://www.dartmouth.edu/~envs/faculty/bolger.html). To date, Wild-ID has been used to accurately identify wildebeest (*Connochaetes taurinus* [20]) and giraffe (*Giraffa camelopardis* [13]). This software uses the SIFT (Scale Invariant Feature Transform) algorithm to characterize variable patterns within photographs and compare all combinations of photographs in a database [21]. SIFT is a convenient pattern-matching algorithm because keypoints are robust to variation in scale and rotation of the photograph [21]. SIFT localizes keypoints within each photo based on local information gradients (Figure 1). Image pairs are then scored and ranked based on the similarity of their keypoint maps. Similarity scores range from 0.0 to 1.0 and provide a standardized measure of pattern resemblance between each image pair. Similarity scores are determined by iteratively comparing geometrically self-consistent subsets of keypoints within pairs of images. During the scoring, each image pair is compared in only one direction (i.e., image A vs. image B, but not B vs. A) and matches are compiled sequentially in the order they were collected (for full details, see [13]).

**Figure 2:**
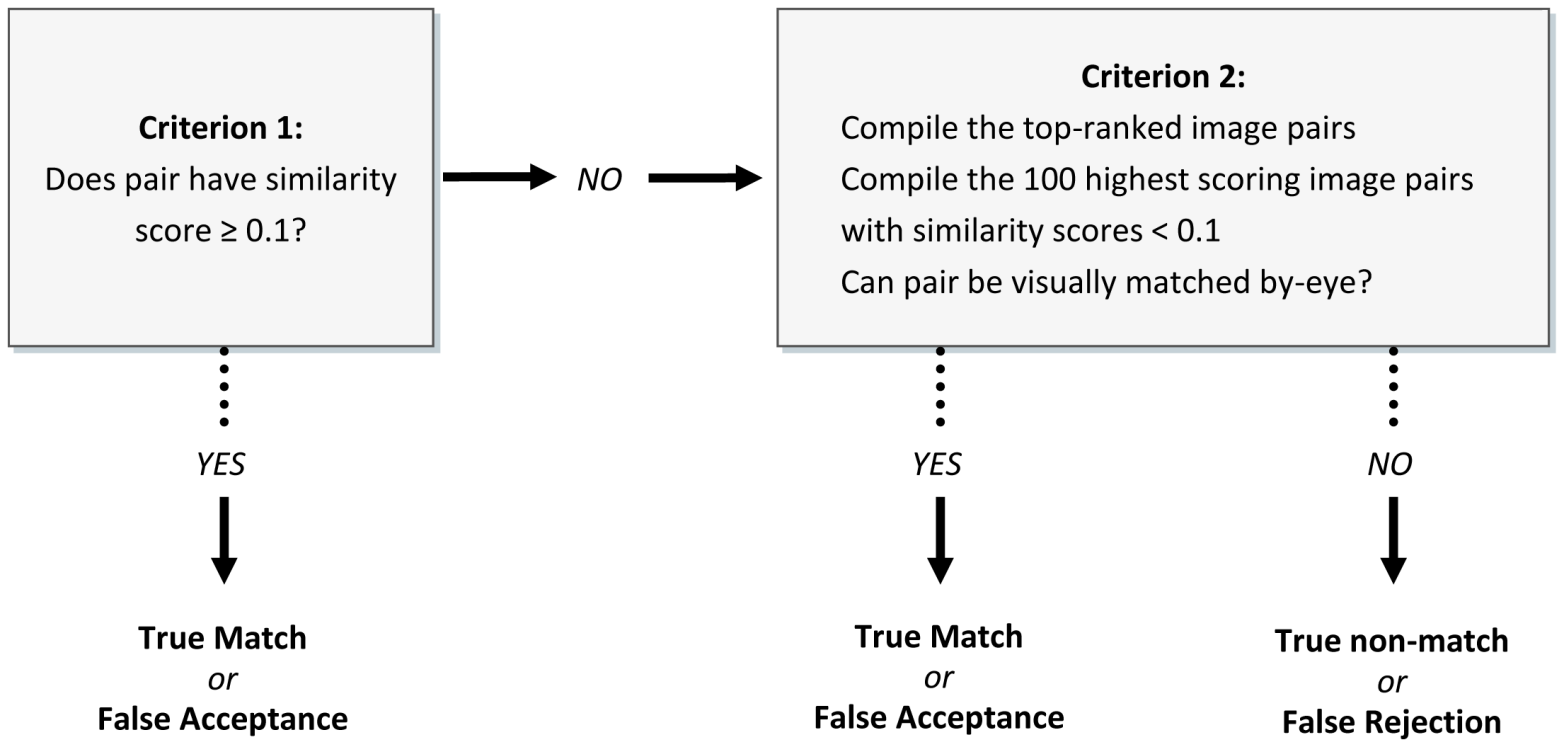
Diagram of the two-step image-matching process. Image pairs above the score threshold of 0.1 were considered matches (criterion 1). The top 100 first-ranked image pairs below 0.1 were compared by eye.

**Figure 3:**
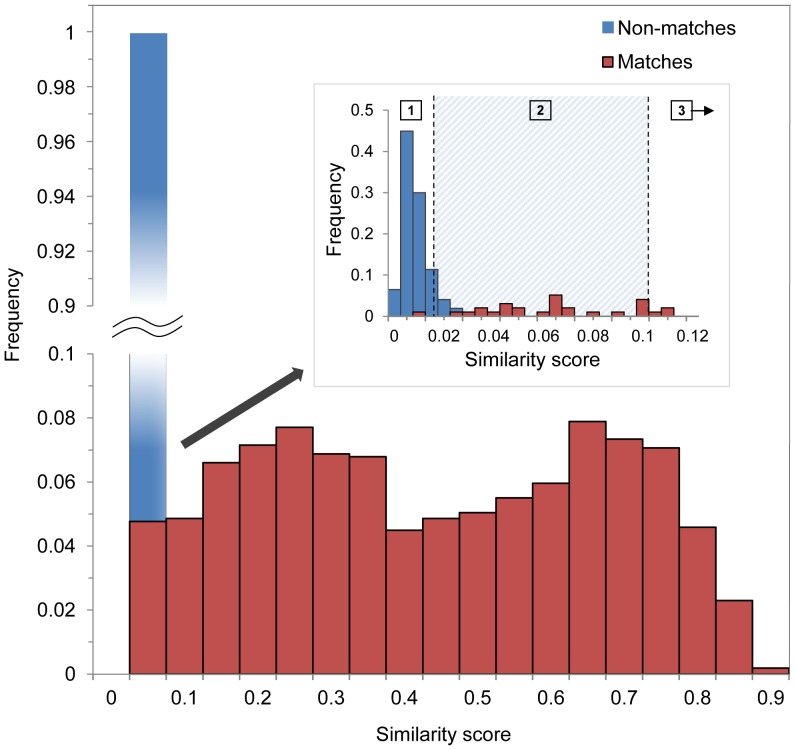
Similarity scores and matching success. Frequency of similarity scores for image pairs from different (black) and the same individuals (grey) from the high-quality dataset. Inset shows the lower range of similarity scores for top ranked image pairs only. The shaded region between dashed lines (2) indicates the range (similarity scores: 0.017–0.1) in which we visually (by-eye) compared 100 potential matching pairs. Similarity scores above this range (3) always involved photo pairs from the same individuals (according to VIE tags). Below this range (1), all but one photo pair came from non-matching individuals (according to VIE tags).

Initial tests using Wild-ID to match photographs of *E. tonkawae* indicated that high scores were exceptionally predictive of correct matches, based on visual confirmation and VIE identification. Thus, we developed a two-step photo matching process to rapidly identify photo matches using Wild-ID (Figure 2). The majority of photo pairs could be accepted or rejected as matches simply based on similarity scores [13]. Examination of the distribution of similarity scores (Figure 3) suggested that scores above 0.1 likely indicated true matches in the high-quality dataset. Thus, we used this threshold as an automated cut-off: images above this threshold were deemed matches, while those below were considered potential matches. Below this threshold score of 0.1 we visually checked the top 100 highest scoring rank-1 image pairs. We chose 100 images for this criterion because it provided an acceptable balance among effort, speed and coverage of likely matches. Based on these two criteria, we generated a capture-history of photos for each unique individual. R scripts [22] used to generate capture histories are included in the supporting information (Files S1 and S2). We used the program ‘Wdump’ to compile scores of all pairwise image comparisons generated by Wild-ID (distributed with Wild-ID, ver. 1.0.1).

Since photograph quality can affect matching success [23], images for matching were segregated into two groups based on camera type: a ‘low-quality’ group (photos taken in 2007 with the point-and-shoot camera) and a ‘high-quality’ group (photos taken between 2008 and 2010 with the DSLR). Images from the two groups were of the same population and included many of the same individuals, but matching occurred only within (and not between) groups. The cameras used to acquire the images differed in resolution (3.14 vs. 10.2 megapixels), format (point-and-shoot vs. DSLR), and spanned several years of technological advances (e.g. release years of 2001 and 2006), resulting in perceptible differences in image quality. Overall, the low-quality dataset suffered from the combined effects of reduced resolution from poorer focus and a more variable subject angle relative to the high-quality dataset. These problems were exacerbated by the use of a lower quality camera, but can also be caused by the photographer’s skill level and factors outside the researcher’s control, such as the subject’s size, speed, and behavior. The incidental use of these two different camera types/technologies in this dataset allowed us to examine the consequences of photo quality on computer-assisted photographic identification.

### Performance of Identification Methods

To assess the accuracy of identification techniques, we calculated two error metrics common to biometric recognition studies: false rejection rates (FRR) and false acceptance rates (FAR) [24]. We define FRR as the frequency of failing to match two captures (either photos or VIE tags) of the same individual:
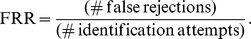



FAR is the frequency of falsely matching two captures (either photos or VIE tags) of different individuals and is calculated as.
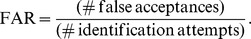



To detect errors in our datasets, we compared capture histories between the two identification methods and manually identified where errors occurred. We first identified VIE errors based on mismatches with the photoID dataset. Importantly, we assumed that visual confirmation of pigmentation patterns in photos provided the baseline for establishing ‘truth’ between two potential matches. For example, if VIEs indicated an incorrect ID for a high-scoring photoID match, we would examine the photos in question to determine whether a photoID false acceptance or a VIE false rejection had occurred. Similarly, if photoID failed to match a VIE pair, we would visually check the photos from the two captures to determine whether the non-match was a VIE false acceptance or a photoID false rejection. We acknowledge the possibility that some errors could have been missed by the combined VIE and photoID identification methods. However, double false acceptance errors should be very infrequent given that we visually confirmed all true matches and pigmentation patterns have high variability between individuals (Figure 1). Further, the double false rejection rate (i.e., FRR_VIE_×FRR_photoID_) and double false acceptance rate (i.e., FAR_VIE_×FAR_photoID_) were extremely low in this study (see Results).

One difficultly we encountered in calculating error rates was that individuals recaptured more than once had multiple, non-independent recapture pairs. Pairs of captures from individuals with large numbers of captures would have been over-represented relative to those with only a few captures. Therefore, we randomly selected a single pair of captures for each unique individual and used this subset of data as the basis for calculating expected error rates. We repeated this subsampling procedure 1000 times and reported the mean error rates across all iterations.

### Performance Over Time

Some natural marking patterns are known to change over time [25], a process that reduces the probability of correctly matching photographs. Therefore, we tested whether the time interval between captures influenced similarity scores in each photo dataset. Again, because some individuals were recaptured on more than one occasion, we used a subsampling procedure that selected a single photo pair per individual. Since many recaptures occur over short time intervals, we made the probability of selecting a particular photo pair proportional to the frequency with which 100-day time intervals were represented in the dataset in order to ensure longer periods were included in subsamples. Given the subsample, we tested whether similarity scores were inversely related to the time interval between captures. We used a Spearman rank test because of heteroscedasticity and lack of normality in similarity scores over time. We repeated the subsampling procedure 1000 times and reported the proportion of iterations in which similarity scores declined significantly over time, where our criterion was P(ρ = 0) <0.01. Additionally, each photo incorrectly rejected by photoID was visually compared to true matches to quantify the mismatches due to either changing natural marks or photo quality. All statistical computations were performed using R [22].

## Results

During 2007, a total of 473 salamanders were marked with VIE, photographed, released, and recaptured (mean no. recaptures = 0.9) over eight primary sampling occasions (24 total surveys), resulting in 965 low-quality photos. Six sampling occasions (18 total surveys) from 2008–2010 resulted in 742 VIE-marked individuals and 1367 high-quality photos (mean no. recaptures = 0.8). None of the 1213 marked salamanders had lost all of their VIE tags, although three individuals were double-marked (i.e. inadvertently marked on two separate occasions) due to VIE misidentification errors. We did not observe any cases where natural marks conflicted with VIE tags when examining image pairs post-hoc to evaluate type and source of error.

VIE misidentification errors were detected by visually examining photoID mismatched image pairs. VIE tags were 2.5-fold more likely to generate false rejections than the high-quality photoID method, and were more likely to generate false acceptances than both photoID datasets (Table 1). FAR and FRR were similar for VIE tags due to the nature of the VIE error-generating process (Table 1). For all VIE false rejections, the mean time to the first misidentification was 10.2 months, or an average of 3.7 primary periods and 1.6 recaptures before an individual’s first VIE misidentification error.

Computer-assisted photoID produced high matching success, particularly in the high-quality dataset. Based on bootstrapped pairwise comparisons, photoID of high-quality photos produced an FRR 20 times lower than the low-quality dataset (Table 1). The FAR was also much higher for the low-quality dataset compared to an FAR of zero for high-quality photos (Table 1). Visual examination of the falsely-rejected photo pairs (57 individuals) revealed that only 5% of photo errors were the result of evolving natural marks, and 11% were the result of both evolving marks and poor photo quality (e.g. blurry or low resolution photos). The remaining errors (84%) were due solely to poor photo quality.

Similarity scores declined over time in both low-quality (*ρ* = −0.31; P<0.01 for 100% of iterations) and high-quality (*ρ* = −0.71; P<0.01 for 100% of iterations) datasets, suggesting that matching performance decreases as the time interval between two matching photographs increases (Figure 3).

## Discussion

One of the most powerful and flexible methods for monitoring animal populations is capture-recapture, a technique that requires accurate identification of individuals over time. Our computer-assisted identification scheme using Wild-ID software exhibited extremely high success for identifying individual salamanders over time with greater accuracy than nearly all other computer-assisted identification studies where error rates have been clearly reported [13]. Scores of rank-1 matches only overlapped rank-1 non-matches marginally (Figure 3) in our high-photo-quality dataset, highlighting the exceptional discriminatory power of score-based identification for *E. tonkawae*. However, false rejection and false acceptance rates increase dramatically when using lower-quality photographs and score-based image matching. Thus, the advantage of semi-automation via score-sorting diminishes when photo quality is poor, requiring visual inspection for more image pairs. Our results suggest that automated photo matching with Wild-ID in which correct matches can be distinguished based on score alone may be suitable for large data sets where photo quality is high. Despite the enormous time-savings compared to manual photo matching [11], computer-assisted identification can still be a time-consuming process for studies that include thousands of images. We suspect the approach using score-based filtering will be most useful to researchers who obtain high-resolution photographs of animals that can be posed with consistent lighting. This technique could be particularly powerful for obtaining rapid (several days of surveying) estimates of population abundance, as it does not require specialized equipment or extensive training.

The predictive ability of Wild-ID scores to correctly identify individuals allowed us to easily cross-check the accuracy of VIE-based matches. Several studies have noted tag loss and migration of VIE tags in amphibians [6], [26], which can violate the capture-recapture assumption that marks do not change over time. Although we did not track retention of individual marks, overall VIE mark retention was generally high, which is consistent with observations from other salamander studies [5], [27], [28]. However, even with high mark retention, VIE read errors and data recording errors can contribute to misidentifications. To reduce identification error, we manually compared photographs of VIE captures and recaptures during data entry to confirm VIE identifications. Despite this, we still encountered instances of inadvertent VIE double-marking and loss/misidentification of more than one VIE mark. These types of errors were extremely difficult to correct during the VIE data validation step (i.e., manual photo-match confirmation) due to the large number of possible matches in our data set. However, these VIE errors were easily identified by computer-assisted photoID. Our analysis revealed that VIE identification had an FRR 2.5 times higher than for photoID. Overall, photoID of *E. tonkawae* resulted in a false rejection rate that was considerably lower than other photoID studies with comparably-sized databases (for review, see [13]). FAR was higher in the VIE dataset than both photoID datasets (Table 1). Unlike false rejections, false acceptance errors do not lead to new unique identifications. Thus, false rejection errors produce relatively greater bias in capture-recapture model estimates [15] and are of greater concern in capture-recapture studies.

Our experience with *Eurycea* salamanders suggests that VIE errors and photoID errors are mostly due to lost or evolving marks, respectively. It is important to note that VIE errors almost always result in false acceptances and false rejections. There is a fixed (known) number of VIE color combinations in any study, and if a novel color combination is observed, the researcher knows that an error occurred. In contrast, there are effectively infinite pigmentation patterns, so evolving patterns typically produce a false rejection but not a false acceptance [11]. Thus, some caution should be taken when comparing error rates across identification methods because their effect on the structure of capture histories, and the way in which errors would be modeled, are different.

Because of high identification accuracy of existing pigmentation patterns, the majority of errors in semi-automated photo-matching for this and similar species are more likely to occur due to evolving natural marks (if photo quality is high). The most common example of evolving natural marks we encountered was due to melanophore expansion and contraction, rendering the overall look of a salamander either lighter or darker. Rapid color changes have been observed in larval *Ambystoma* [29] and we documented individuals exhibiting dramatic changes in melanophore size within 24 hours. Patterns also change due to growth, development and gravidity [7]. For example, melanophores in young individuals expand during growth, making it difficult to recognize individuals that have considerably changed size since their last observation. Despite these potential challenges, error rates were still low when using high-quality images across all time intervals.

**Figure 4:**
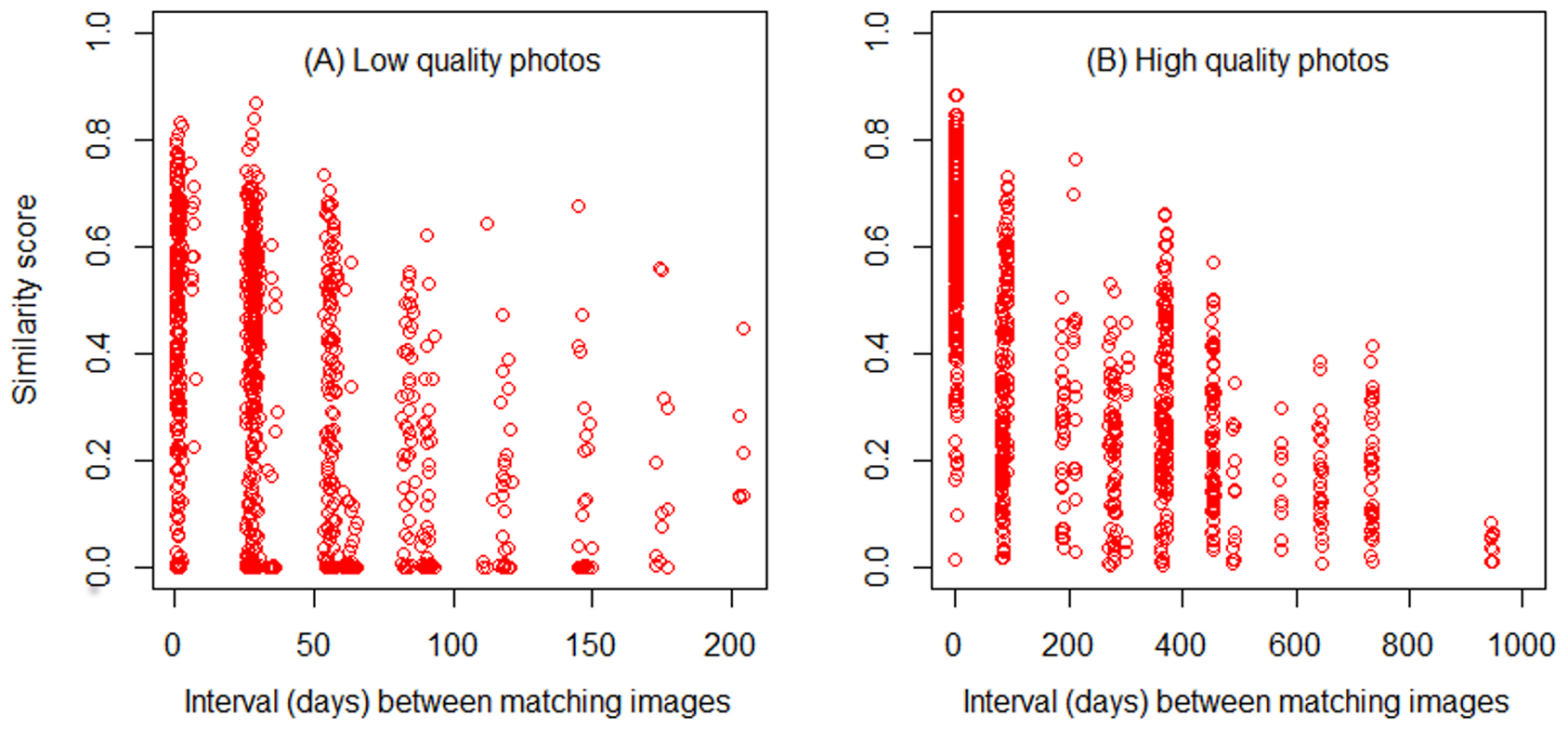
Effect of time on similarity score. Similarity scores versus days between photo captures of the same individual for (A) low-quality (*n* = 896) and (B) high-quality photos (*n* = 1090). Tables.

Although evolving natural marks did not significantly inhibit the accuracy of photoID of *E. tonkawae* during this four-year study, there was a negative relationship between similarity scores of matching pairs and the time interval between successive captures (Figure 4). Our results suggest matching success remains high, even across long intervals, as long as image quality is high. Lower quality matching image pairs have lower similarity scores over time and likely generate higher error rates. Species whose natural marks change quickly over time, either seasonally or annually, will require relatively short intervals between capture periods and high capture rates within periods to maintain sufficiently low error rates. Thus, the relationship between misidentification rate and time interval between captures depends on (1) how quickly marks evolve between successive captures, and (2) on the quality of photographs.

Additional optimization of the identification software and photo-collection methods could have conceivably improved our matching success. Given the low initial error rates observed with Wild-ID, we did not test other software platforms nor attempt to further optimize the Wild-ID scoring algorithm. Photo ID applications aimed at species with more challenging natural marking patterns [20] or species with evolving marks [19] may require either optimization of the Wild-ID scoring algorithm [13] or modeling of the error process. Indeed, new methods are already being developed to accommodate photoID errors in capture-recapture modeling [30] including errors involving evolving natural marks [16].

The use of photo-only based identification for *Eurycea tonkawae* has a number of advantages over VIE based approaches in addition to substantial reductions in false rejection and false acceptance rates. PhotoID capture-recapture is (1) less invasive, as it does not require injections or anaesthetization; (2) faster, both in terms of time required in the field to mark individuals and time in the office to process data; (3) less expensive, since it requires less time and overall equipment costs are lower for large studies (camera setup has high initial cost, but VIE consumables are expensive and frequently need replacement); and (4) requires less experience, since it is easier to produce a quality photograph than to inject VIE tags into a small salamander in the field. Photographic identification with Wild-ID shows considerable promise as a substitute for VIE marking in spring-dwelling *Eurycea*, and potentially other taxa as well.

### Conservation Implications

The development of an inexpensive, fast, accurate and relatively non-invasive method for tracking *Eurycea* is timely. Four salamander species (*E. chisholmensis*, *E. naufragia*, *E. tonkawae* and *E. waterlooensis*) endemic to central Texas were recently proposed for protection under the Endangered Species Act of 1973, highlighting the need for a better understanding of their ecology [17]. Until recently, most published research on central Texas *Eurycea* has focused on their unique morphological variation [31]–[34], physiology [35], [36], distribution [37] and taxonomy [18], [38], [39]. Despite ample scientific interest and numerous anthropogenic threats facing these species [40], surprisingly few studies have focused on understanding the population dynamics, life history or dispersal of any of the central Texas *Eurycea* salamanders (though see [41], [42]). These facets of the salamanders’ ecology are central to conservation efforts and largely require individual-level identification. While recent research is beginning to shed light on the ecology of spring-dwelling *E. naufragia* [43], [44] and *E. tonkawae* [19], [45], much remains to be learned about the ecology and population dynamics of these and other central Texas *Eurycea*. The advantages of photography-based identification over VIE marking, as demonstrated here, may facilitate an expansion of ecological knowledge about these endangered, karst-dwelling species that will ultimately help guide conservation efforts and sound management practices.

## Supporting Information

File S1 Wdump scores to capture history R code.
(TXT)

File S2 Wild-ID confirmed matches to capture history R code.
(TXT)
